# Diagnostic models for fever of unknown origin based on ^18^F-FDG PET/CT: a prospective study in China

**DOI:** 10.1186/s13550-022-00937-4

**Published:** 2022-10-28

**Authors:** Jia Chen, MingYou Xing, Dong Xu, NaNa Xie, WenCong Zhang, QiuRong Ruan, JianXin Song

**Affiliations:** 1 Department of Infectious Diseases, Tongji Hospital, Tongji Medical College, Huazhong University of Science and Technology, 1095 Jiefang Avenue, Wuhan, 430030 China; 2 Institute of Pathology, Tongji Hospital, Tongji Medical College, Huazhong University of Science and Technology, 1095 Jiefang Avenue, Wuhan, 430030 China

**Keywords:** FUO, ^18^F-FDG PET/CT, Clinical parameters, Diagnostic model

## Abstract

**Background:**

This study aims to analyze the ^18^F-fluorodeoxyglucose positron emission tomography/computed tomography (^18^F-FDG PET/CT) characteristics of different causes of fever of unknown origin (FUO) and identify independent predictors to develop a suitable diagnostic model for distinguishing between these causes. A total of 524 patients with classical FUO who underwent standard diagnostic procedures and PET/CT were prospectively studied. The diagnostic performance of PET/CT imaging was analyzed, and relevant clinical parameters that could improve diagnostic efficacy were identified. The model was established using the data of 369 patients and the other 155 patients comprised the validation cohort for verifying the diagnostic performance of the model.

**Results:**

The metabolic characteristics of the “hottest” lesion, the spleen, bone marrow, and lymph nodes varied for various causes. PET/CT combined with clinical parameters achieved better discrimination in the differential diagnosis of FUO. The etiological diagnostic models included the following factors: multisite metabolic characteristics, blood cell counts, inflammatory indicators (erythrocyte sedimentation rate, C-reactive protein, serum ferritin, and lactate dehydrogenase), immunological indicators (interferon gamma release assay, antinuclear antibody, and anti-neutrophil cytoplasm antibody), specific signs (weight loss, rash, and splenomegaly), and age. In the testing cohort, the AUCs of the infection prediction model, the malignancy diagnostic model, and the noninfectious inflammatory disease prediction model were 0.89 (95% CI 0.86–0.92), 0.94 (95% CI 0.92–0.97), and 0.95 (95% CI 0.93–0.97), respectively. The corresponding AUCs for the validation cohort were 0.88 (95% CI 0.82–0.93), 0.93 (95% CI 0.89–0.98), and 0.95 (95% CI 0.92–0.99), respectively.

**Conclusions:**

^18^F-FDG PET/CT has a certain level of sensitivity and accuracy in diagnosing FUO, which can be further improved by combining it with clinical parameters. Diagnostic models based on PET/CT show excellent performance and can be used as reliable tools to discriminate the cause of FUO.

*Trial registration* This study (a two-step method apparently improved the physicians’ level of diagnosis decision-making for adult patients with FUO) was registered on the website http://www.clinical-trials.gov on January 14, 2014, with registration number NCT02035670.

**Supplementary Information:**

The online version contains supplementary material available at 10.1186/s13550-022-00937-4.

## Introduction

Fever of unknown origin (FUO) was originally described by Petersdorf and Beeson as a temperature repeatedly higher than 38.3 °C for at least 3 weeks with no ascertainable cause after a week of hospitalization and investigation [1]. FUO remains a substantial challenge for clinicians due to its inclusion of approximately 200 potential causes in four categories: infections, noninfectious inflammatory diseases (NIIDs), malignancies, and miscellaneous causes [2–6]. The pathophysiology, treatment, and prognosis of these categories differ markedly [7]. Early identification of the underlying category is important to optimize treatment strategies for patients with FUO [8–10]. Currently, ^18^F-fluorodeoxyglucose positron emission tomography/computed tomography (^18^F-FDG PET/CT) is considered one of the most promising diagnostic tools for FUO [11–29].

Previous studies have mainly been small case series (between 24 and 376 cases) and retrospective studies [11–21]. The application of PET/CT in etiological classification of FUO was not sufficient. Although PET/CT can provide some diagnostic clues, it is difficult to directly provide a definitive etiological diagnosis of FUO. Therefore, we conducted a study in a Chinese population to explore the PET/CT characteristics for different causes of FUO and further evaluate the clinical significance of PET/CT in the diagnosis of FUO. Specifically, the purpose of this study was to identify the most suitable model for diagnosing patients with FUO based on PET/CT imaging.

## Methods

This study aims to analyze the PET/CT characteristics of different causes of FUO and identify independent predictors to develop a suitable diagnostic model for distinguishing between these causes.

### Patients and standard diagnostic work-up

This prospective study recruited patients older than 14 years of age with a diagnosis of classical FUO who were hospitalized at Tongji Hospital in Wuhan, Hubei Province, China, from January 2016 to July 2021, finally including 369 patients admitted to the Department of Infectious Diseases and 155 patients admitted to other departments (i.e., the Department of Hematology, the Department of Rheumatology, and the Department of Pulmonary and Critical Care Medicine). All enrolled patients underwent a defined standard diagnostic work-up and ^18^F-FDG PET/CT scans when there were no potential diagnostic clues (PDCs). Classical FUO was defined as follows: (1) an illness of more than 3 weeks in duration, (2) temperature exceeding 38.3 °C on more than three occasions, and (3) an uncertain diagnosis despite appropriate investigation, with at least three outpatient visits or at least 3 days of hospitalization [5]. The exclusion criteria were as follows: (1) nosocomial FUO, (2) immunodeficiency-associated FUO, and (3) travel-associated FUO. All enrolled patients were followed up within 6 months via telephone. This study on FUO was registered on the website http://www.clinical-trials.gov on January 14, 2014, with registration number NCT02035670. The research protocol was reviewed and approved by the Huazhong University of Science and Technology Clinical Trial Ethics Committee ([2014] EC-No. 16). Written informed consent was obtained from all participants.

Standard diagnostic procedures included the first step: a thorough patient history questionnaire (a written medical history questionnaire from a previous study), careful physical examination, and obligatory investigation (e.g., hemoglobin, platelet count, leukocyte count and differentiation, creatine, total protein, protein electrophoresis, aspartate aminotransferase, alanine aminotransferase, erythrocyte sedimentation rate [ESR], C-reactive protein, lactate dehydrogenase [LDH], serum ferritin [SF], procalcitonin, antinuclear antibody [ANA], anti-neutrophil cytoplasm antibody [ANCA], interferon gamma release assay [IGRA], blood culture [n = 3], urine culture, chest CT, and ultrasound) [15, 30]. When PDCs were absent, PET/CT was performed, and further confirmatory procedures (e.g., biopsy, culture, response to diagnostic therapy) were selected based on the results. Changes in relevant blood indicators were monitored, and their dynamic processes were recorded. The final clinical diagnosis of each patient was established by the study team based on the results of all standard procedures, including laboratory, radiological, histopathological findings, and clinical course, according to relevant disease diagnosis guidelines. It was further confirmed by follow-up for 6 months. The causes were divided into five categories: infection, NIID, malignancy, miscellaneous cause, and unknown cause.

### PET/CT scans

The PET/CT scanner used in this study was a Discovery PET/CT 690 (GE Healthcare). PET/CT scans were acquired using standard techniques based on the guidelines issued by the Chinese Society of Nuclear Medicine (fasting for at least 6 h, blood glucose levels below 11.1 mmol/l, and imaging performed 1 h after injection of 2.96–4.44 MBq/kg of FDG) [31]. Emission and transmission images of the area between the proximal femur and the base of the skull were obtained. Attenuation correction and image reconstruction were carried out with an iterative method. Lesion FDG uptake was quantitatively evaluated by using the maximum standardized uptake value (SUVmax). PET/CT images were independently evaluated by two experienced nuclear medicine physicians who were blinded to the detailed clinical data. Any disagreements were resolved by consensus.

### Statistical analysis

Statistical analysis was performed using SPSS Statistics 26.0 Software (IBM, Chicago). Data are presented as the mean ± SD, median [IQR], or frequencies (percentage), when appropriate. Comparisons of continuous variables between groups were performed with Student’s *t* test or the Mann–Whitney U test depending on the data distribution. Categorical variables were compared between groups using the χ^2^ or Fisher’s exact test according to the theoretical frequency. Two-sided p values < 0.05 were considered to indicate significance.

Univariate analysis was used to screen potential diagnostic indicators. All candidate variables with p values < 0.20 in the univariate analysis were included in a multivariate analysis to identify the indexes eligible for inclusion in the model. The significance level was set at p < 0.05 in the multivariate analysis. The results of the multivariate analysis are presented as odds ratios (ORs) and 95% confidence intervals (CIs). The area under the receiver operating characteristic curve (AUC) was used to evaluate the performance of the prediction model.

## Results

### Patients’ characteristics and diagnoses

Overall, 524 FUO patients were enrolled in this study, including 285 males (285/524, 54.4%), with a median age of 49 years [32–61 years]. The duration of fever ranged from 21 to 732 days, with a median of 37 days. Infectious diseases, tumors, NIIDs, and miscellaneous causes were reported in 223, 121, 109, and 22 patients, respectively, while the remaining 49 patients were undiagnosed after 6 months of follow-up. The final etiological classifications and clinical diagnoses are listed in Additional file 1: Table S1. Tuberculosis accounted for the largest proportion of infections (52/223, 23.3%), while lymphoma was the most common tumor (77/121, 63.6%). Half of the NIID patients (56/109, 51.4%) had adult-onset Still’s disease. There was no significant difference in etiological composition or patient characteristics between patients from the Department of Infectious Diseases and those from other departments (Table 1).

**Table 1:** Characteristics of individuals and etiological composition between patients from different departments

	Patients from the department of infectious diseases (n = 369)	Patients from other departments (n = 155)	p value
Sex male	193 (52.3)	92 (59.4)	0.139
Age (years)	48 [32–60]	50 [32–62]	0.315
Duration of fever (days)	37 [28–57]	38 [28–65]	0.564
Infection	157 (42.5)	66 (42.6)	0.969
Malignancy	85 (23.0)	36 (23.2)	
NIID	76 (20.6)	33 (21.3)	
Miscellaneous cause	17 (4.6)	5 (3.2)	
Unknown cause	34 (9.2)	15 (9.7)	

N (%), median [IQR]

*NIID* noninfectious inflammatory disease

### PET/CT characteristics and performance in diagnosing FUO

**Fig. 1:**
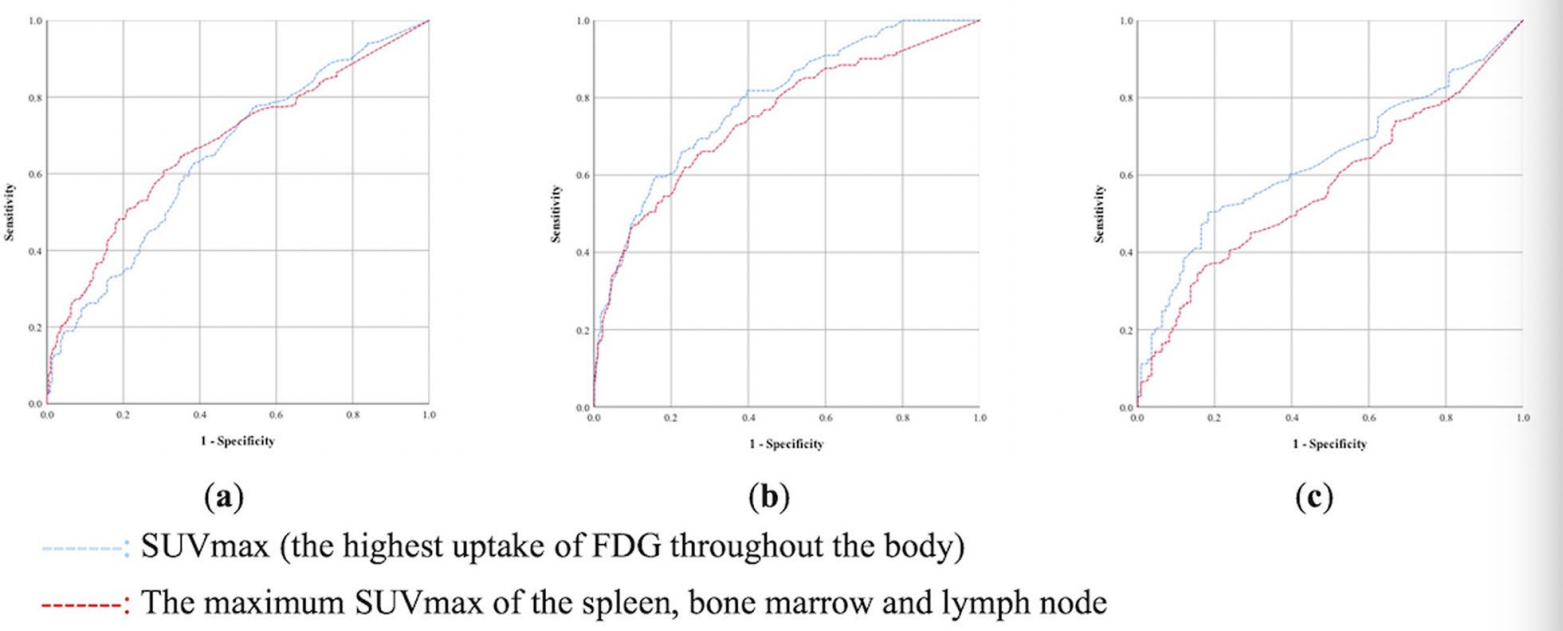
**a** ROC curve of SUVmax and the maximum SUVmax of the spleen, bone marrow, and lymph node in diagnosing infection. **b **ROC curve of SUVmax and the maximum SUVmax of the spleen, bone marrow, and lymph node in diagnosing malignancy. **c** ROC curve of SUVmax and the maximum SUVmax of the spleen, bone marrow, and lymph node in diagnosing NIID

PET/CT examinations showed positive findings in 477 (477/524, 91.0%) patients (diffuse or focal high uptake of FDG in various organs and tissues) (Table 2). All patients with neoplasms had positive results. The locations of the lesions with the highest FDG uptake in different etiologies were listed in Additional file 1: Table S2. Lymph nodes and bone marrow were the most common sites with the highest uptake among all etiologies. Hyperenhancement of the nasopharynx was common in tumors, and hyperenhancement of the bone or joint was common in inflammation. The SUVmax for the “hottest” lesions was significantly higher in patients with malignancy than that in patients with infections and NIIDs (p < 0.05). The AUC of SUVmax was 0.79 (95% CI 0.74–0.84) in diagnosing cancer, 0.65 (95% CI 0.60–0.70) in diagnosing infection, and 0.64 (95% CI 0.59–0.69) in diagnosing NIID (Fig. 1).

**Table 2:** Positive findings and SUVmax of PET/CT for patients with FUO

PET/CT findings	Infection	Malignancy	NIID	Miscellaneous	Unknown	p value
Positive	193 (86.5)^a^	121 (100)^b^	99 (90.8)^a^	22 (100)^a, b^	42 (85.7)^a^	0.000
SUVmax	5.4 [4.0, 9.0]^a, c^	10.8 [6.7, 16.4] ^b^	5.1 [3.9, 6.5]^a^	8.3 [5.5, 12.3]^b, c^	7.8 [5.8, 9.1]^b^	0.000

N (%), median [IQR]

*SUVmax* the maximum standardized uptake value; *PET/CT* positron emission tomography/computed tomography; *FUO* fever of unknown origin; *NIID* noninfectious inflammatory disease

^a, b, c^Pairwise comparison between subgroups, p values < 0.05

Many patients showed FDG avidity in multiple sites, most commonly in the spleen, bone marrow, and lymph nodes (Table 3). The metabolic characteristics of the spleen, bone marrow, and peripheral lymph nodes (cervical, axillary, and inguinal lymph nodes) were consistent. Specifically, the increased FDG uptake was more common, and the corresponding SUVmax was higher in patients with tumors, while related indexes were significantly decreased in patients with infectious diseases (p < 0.05). Patients with NIIDs presented with high rates of hypermetabolism, similar to those with malignancies, but a lower corresponding SUVmax than patients with malignancies (p < 0.05). The metabolic characteristics of the central lymph nodes were different. The incidence of increased FDG avidity in the retroperitoneal lymph nodes in NIID patients was lower than that in patients with tumors (p < 0.05). There was no significant difference in the proportion of mediastinal lymph node hypermetabolism among the groups. Further analysis confirmed that the metabolic characteristics of the spleen, bone marrow and lymph nodes performed modestly in categorizing FUO etiology into infection, malignancy, and NIID, and the AUCs of the above indicators were mostly less than 0.7 (Additional file 1: Fig. S1). The maximum SUVmax of the spleen, bone marrow, and lymph node yielded an AUC of 0.75 (95% CI 0.69–0.80) in diagnosing cancer, 0.68 (95% CI 0.63–0.72) in diagnosing infection, and 0.57 (95% CI 0.52–0.63) in diagnosing NIID (Fig. 1).

**Table 3:** Increased FDG uptake in the spleen, bone marrow, and lymph nodes for patients with FUO

Location	Characteristics	Infection	Malignancy	NIID	Miscellaneous	Unknown	p value
Spleen	Hypermetabolism	50 (22.4) ^a^	64 (52.9)^b^	50 (45.9)^b^	5 (22.7)	27 (55.1)	0.000
	SUVmax	3.6 [3.0, 4.3]^a^	5.2 [3.6, 7.8]^b^	3.9 [3.3, 5.0]^a^	3.0 [2.7, 4.4]	4.3 [3.4, 5.5]	0.000
bone marrow	Hypermetabolism	48 (21.5)^a^	53 (43.8)^b^	41 (37.6)^b^	4 (18.2)	18 (36.7)	0.000
	SUVmax	4.4 [3.5, 5.1]^a^	6.6 [4.4, 10.2]^b^	4.4 [3.7, 5.4]^a^	5.5 [4.2, 6.8]	4.6 [3.7, 5.7]	0.000
Lymph node	Hypermetabolism	149 (66.8)^a^	95 (78.5) ^a^	74 (67.9)^a^	18 (81.8)	33 (67.3)	0.129
	SUVmax	4.0 [2.9, 6.4]^a^	9.1 [4.4, 14.3] ^b^	4.3 [2.8, 6.4]^a^	8.7 [4.6, 12.3]	7.4 [5.6, 9.0]	0.000
Cervical LN	Hypermetabolism	83 (37.2)^a^	66 (54.5)^b^	57 (52.3)^b^	16 (72.7)	26 (53.1)	0.001
	SUVmax	3.5 [2.4, 5.6]^a^	5.6 [3.0, 13.5)^b^	3.8 [2.5, 5.3]^a^	8.2 [4.2, 12.6]	6.0 [4.0, 8.0]	0.000
Axillary LN	Hypermetabolism	41 (18.4)^a^	40 (33.1)^b^	48 (44.0)^b^	8 (36.4)	23 (46.9)	0.000
	SUVmax	2.7 [1.9, 4.3]^a^	5.3 [2.4, 12.4] ^b^	3.3 [1.9, 5.1]^a^	7.7 [4.1, 10.5]	6.5 [2.9, 8.1]	0.000
Inguinal LN	Hypermetabolism	19 (8.5)^a^	28 (23.1)^b^	26 (23.9)^b^	7 (31.8)	11 (22.4)	0.000
	SUVmax	2.9 [1.7, 3.6]^a^	5.0 [2.3, 14.2]^b^	2.9 [1.9, 4.4]^a^	2.9 [1.4, 4.8]	3.1 [1.8, 5.4]	0.021
Mediastinal LN	Hypermetabolism	95 (42.6)^a^	58 (47.9)^a^	43 (39.4)^a^	4 (18.2)	18 (36.7)	0.101
	SUVmax	3.9 [2.9, 5.2]^a^	5.8 [3.2, 14.0]^b^	4.2 [3.0, 6.7]^a, b^	5.0 [3.5, 6.7]	6.0 [3.5, 7.6]	0.008
Retroperitoneal LN	Hypermetabolism	53 (23.8)^a^	66 (54.5)^b^	33 (30.3)^a^	10 (45.5)	23 (46.9)	0.000
	SUVmax	3.7 [2.6, 6.8]^a^	9.7 [5.2, 14.6]^b^	4.3 [3.3, 6.0]^a^	7.4 [5.2, 9.0]	5.2 [3.6, 7.7]	0.000

N (%), median [IQR]

*FDG* fluorodeoxyglucose; *FUO* fever of unknown origin; *NIID* noninfectious inflammatory disease; *SUVmax* the maximum standardized uptake value; *LN* lymph node

^a, b^ Pairwise comparison between subgroups, p values < 0.05

### Diagnostic model construction

Due to the small number of patients with no diagnosis and miscellaneous diseases, the lack of commonality among those diseases, and the desire to use all the data to meet ﻿real-life settings, three categories were adopted: infectious disease and noninfectious disease; malignant disease and nonmalignant disease; and NIID and non-NIID. A clinical prediction model was established based on the data of 369 patients from the Department of Infectious Diseases.

PET/CT features and clinical parameters, including patient general characteristics, specific signs, blood cell counts, biochemical indexes, and immunological indicators, were included in the screening. Relevant blood indicators included not only the values measured at admission, but also the maximum and minimum values over the course of disease. Beneficial predictors that contributed to improving diagnostic efficacy were identified. For each significant independent variable, a more detailed analysis was performed (Table 4).

**Table 4:** Potential diagnostic indicators and results of the logistic regression for infection, malignancy, and NIID models

Categories	Characteristics	Univariate analysis		Multivariate analysis	
		OR (95% CI)	p value	OR (95% CI)	p value
Infection	Low SUVmax of spleen, bone marrow, and lymph nodes	4.21 (2.63–6.76)	< 0.01	2.18 (1.31–3.65)	0.01
	Low SUVmax of liver ratio	2.19 (1.57–3.04)	< 0.01	2.52 (1.25–5.09)	< 0.01
	IGRA positive	6.46 (3.20–13.05)	< 0.01	13.09 (4.83–35.44)	< 0.01
	ANA and ANCA negative	13.27 (3.13–56.36)	< 0.01	20.23 (4.36–93.82)	< 0.01
	Low ESR	3.88 (2.04–7.40)	< 0.01	8.04 (3.45–18.75)	< 0.01
	High platelet	4.43 (2.10–9.37)	< 0.01	5.84 (2.10–16.23)	< 0.01
	Low neutrophilic percentage	4.06 (2.33–7.08)	< 0.01	3.62 (1.77–7.38)	< 0.01
	Low LDH	4.04 (2.61–6.27)	< 0.01	3.46 (1.83–6.53)	< 0.01
	Rash negative	2.80 (1.59–4.93)	< 0.01	2.81 (1.30–6.07)	< 0.01
Malignancy	SUVmax	1.25 (1.18–1.32)	< 0.01	1.34 (1.24–1.46)	< 0.01
	LDH /100	1.16 (1.09–1.22)	< 0.01	1.30 (1.16–1.46)	< 0.01
	Low SF	1.58 (0.82–3.04)	0.17	9.89 (2.87–34.09)	< 0.01
	High nasopharynx SUVmax	8.37 (3.07–22.77)	< 0.01	6.59 (1.59–27.29)	< 0.01
	Weight loss	4.00 (2.34–6.83)	< 0.01	6.19 (2.70–14.22)	< 0.01
	Low platelet	6.10 (3.66–10.16)	< 0.01	5.96 (2.72–13.05)	< 0.01
	Splenomegaly	6.12 (3.53–10.62)	< 0.01	3.91 (1.64–9.32)	< 0.01
NIID	ANA or ANCA positive	12.28 (5.53–27.29)	< 0.01	64.74 (18.74–223.65)	< 0.01
	Low SUVmax of liver ratio	6.55 (2.91–14.76)	< 0.01	15.32 (4.26–55.03)	< 0.01
	High platelet	3.19 (1.85–5.47)	< 0.01	9.75 (3.65–26.03)	< 0.01
	Rash positive	4.58 (2.63–7.97)	< 0.01	11.06 (4.33–28.28)	< 0.01
	High SF	4.12 (2.36–7.18)	< 0.01	7.02 (2.69–18.27)	< 0.01
	High neutrophilic percentage	4.43 (2.88–6.83)	< 0.01	6.69 (3.43–13.06)	< 0.01
	Young	2.56 (1.53–4.29)	< 0.01	3.03 (1.34–6.86)	< 0.01

*NIID* noninfectious inflammatory disease; *OR* odds ratio; *CI* confidence interval; *ESR* erythrocyte sedimentation rate; *LDH* lactate dehydrogenase; *SF* serum ferritin; *SUVmax* the maximum standardized uptake value; *IGRA* interferon gamma release assay; *ANA* antinuclear antibody; *ANCA* anti-neutrophil cytoplasm antibody

**Fig. 2:**
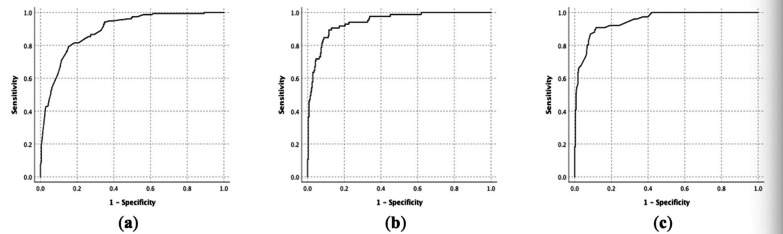
**a** ROC curve of the infection prediction model. **b** ROC curve of the malignancy prediction model. **c** ROC curve of the NIID prediction model

Factors with higher OR values for diagnosing infection included: low SUVmax (the “hottest” lesion, spleen, bone marrow, and lymph nodes), high platelet count, low neutrophilic percentage, low ESR, low LDH, positive IGRA, negative ANA and ANCA, and negative rash (Table 4). The probability of infection could be calculated according to the formula in Table 5. The AUC was 0.89 (95% CI 0.86–0.92) (Fig. 2). When the optimal cutoff point was 0.46, the sensitivity, specificity, positive predictive value (PPV), and negative predictive value (NPV) were 81.5%, 81.6%, 76.4%, and 85.4%, respectively.

**Table 5:** Formulas for the Infection, malignancy, and NIID prediction model

Categories	Formula
Infection prediction model	logit (p) = 0.92* (maximum SUVmax of spleen, bone marrow and lymph node < 5.7 = 1) + 0.78* (2.3 ≤ SUVmax of liver ratio < 4.7 = 1, SUVmax of liver ratio < 2.3 = 2) + 1.76* (maximum platelets > 132*10^9^/L = 1) + 1.29* (minimum neutrophilic percentage < 65% or maximum neutrophilic percentage < 85% = 1) + 2.09* (ESR ≤ 96 mm/H = 1) + 1.24* (maximum LDH ≤ 340 U/L = 1) + 2.57*IGRA (positive = 1) + 3.01*ANA and ANCA (both negative = 1) + 1.03*rash (negative = 1)− 10.17
Malignancy prediction model	0.29*SUVmax + 1.89* (nasopharynx SUVmax ≥ 5.6 = 1) + 1.79* (35*10^9^/L < platelet ≤ 170*10^9^/L = 1, platelet < 35*10^9^/L = 2) + 0.26*LDH/100 + 2.29* (SF < 3200 ug/L = 1) + 1.36*splenomegaly (splenic thickness ≥ 4.7 cm = 1) + 1.82* (weight loss = 1)–8.64
NIID prediction model	logit (p) = 2.73* (SUVmax of liver ratio ≤ 2.8 = 1) + 2.28* (platelet > 210*10^9^/L = 1) + 1.9* (maximum neutrophilic percentage ≥ 85.7%, minimum neutrophilic percentage ≥ 52.5%, both positive = 2, either positive = 1) + 1.95* (SF ≥ 3200 ug/L = 1) + 4.17*ANA and ANCA (either positive = 1) + 2.4*rash (positive = 1) + 1.11*young (age ≤ 43 years = 1)−9.8

*NIID* noninfectious inflammatory disease; *SUVmax* the maximum standardized uptake value; *ESR* erythrocyte sedimentation rate; *LDH* lactate dehydrogenase; *IGRA* interferon gamma release assay; *ANA* antinuclear antibody; *ANCA* anti-neutrophil cytoplasm antibody; *SF* serum ferritin

The model for predicting malignancy included the following parameters: high SUVmax (the “hottest” lesion and nasopharynx), low platelet count, high LDH, low SF, weight loss, and splenomegaly (Table 4). The probability of malignancy could be calculated according to the formula in Table 5. The AUC was 0.94 (95% CI 0.92–0.97) (Fig. 2). When the optimal cutoff point was 0.19, the sensitivity, specificity, PPV, and NPV were 90.6%, 87.0%, 71.8%, and 94.0%, respectively.

The model for predicting NIID was composed of low SUVmax (the “hottest” lesion), high platelet count, high neutrophilic percentage, high SF, positive ANA or ANCA, positive rash, and young age (Table 4). The probability of NIID could be calculated according to the formula in Table 5. The AUC was 0.95 (95% CI 0.93–0.97) (Fig. 2). When the optimal cutoff point was 0.21, the sensitivity, specificity, PPV, and NPV were 90.8%, 88.4%, 75.0%, and 93.5%, respectively.

### Diagnostic model validation

A total of 155 patients from other departments comprised an independent external validation cohort. The proportion of patients in the training and validation data sets was close to 7:3, as previously described [32, 33].

**Fig. 3:**
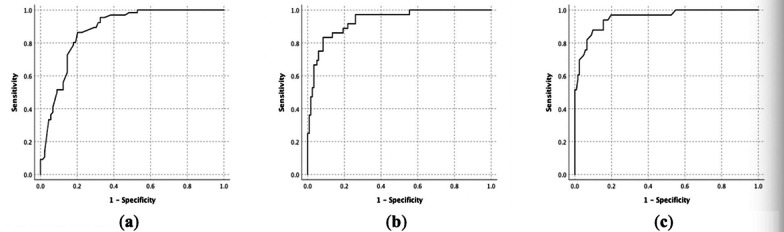
**a** ROC curve of the infection model in the validation cohort. **b** ROC curve of the malignancy model in the validation cohort. **c** ROC curve of the NIID model in the validation cohort

According to the above models, the probabilities of the corresponding diseases in the validation cohort were calculated, and the accuracies were further analyzed (Fig. 3). In the validation cohort, the AUC of the infectious disease model was 0.88 (95% CI 0.82–0.93), and when the cutoff point was 0.46, the model sensitivity and specificity were 86.4% and 79.8%, respectively. The AUC of the malignancy model in the validation cohort was 0.93 (95% CI 0.89–0.98). When the cutoff point was 0.19, the model sensitivity was 86.1%, and the specificity was 85.7%. The AUC for validation of the NIID model in the cohort was 0.95 (95% CI 0.92–0.99), and when the cutoff point was 0.21, the model sensitivity was 90.9%, and the specificity was 84.4%.

## Discussion

Due to the complexity and diversity of FUO-related diseases, the multiplicity of etiologies, the atypical symptoms, and the small number of diagnostic clues, diagnosing the intrinsic etiologies of FUO remains a challenge for contemporary clinicians, requiring extensive time-consuming physical and laboratory tests and even diagnostic treatment. In this study, infection (223/524, 42.6%) accounted for the largest proportion of cases. A total of 23.1% (121/524) of patients had malignancies, 20.8% (109/524) had NIIDs, and 9.4% (49/524) received no diagnosis. This etiological distribution is in concordance with a Chinese survey involving 1641 patients with FUO [34]. Early identification of the underlying cause and implementation of appropriate empirical treatment are effective strategies for patients with FUO.

The high sensitivity and wide visual field of PET/CT are helpful in detecting lesions that are clinically unknown or undetectable with conventional imaging techniques and in obtaining multisystem observations [25, 26]. FDG uptake is an indicator of increased intracellular glucose metabolism, and this molecule can be absorbed not only by malignant cells, but also by cells involved in infection and inflammation [13, 14, 18, 19, 25]. The presence of abnormal uptake can help guide further investigations, leading to a definitive diagnosis. The absence of abnormal absorption reasonably ensures that the possible underlying conditions are not present, thus avoiding unnecessary additional testing. Therefore, in recent years, the diagnostic role of PET/CT in FUO has been widely recognized, especially in oncology [11–19]. SUVmax can be obtained by routine scanning, which is easy to obtain and reproduce and directly reflects the highest activity of the lesion, so it is widely used in clinical practice. Consistent with previous studies, the uptake of FDG in neoplastic diseases was significantly higher than that in other diseases. SUVmax had moderate diagnostic performance for malignancy with an AUC of 0.79 and a low diagnostic performance for nonneoplastic diseases, which is not satisfactory for clinicians. Although the lymph nodes and bone marrow were the most common sites of the highest enhancement in all etiologies, other hyperenhancement sites varied among etiologies. In particular, nasopharyngeal hypermetabolism was common in tumors; the final diagnostic model confirmed that nasopharyngeal metabolic characteristics were helpful for the diagnosis of tumors.

Previous studies divided positive PET/CT results into focal FDG uptake and nonspecific abnormal uptake (NAU). NAU manifests as spleen and bone marrow diffuse high uptake and multiple reactive hyperplasia lymph nodes with high uptake and symmetrical distribution. NAU is common and has been considered a negative or a minor factor in diagnosis in some studies [12–15, 17, 18]. However, there were significant differences in the metabolic characteristics of the spleen, bone marrow, and lymph nodes among different etiologies of FUO. In general, infection manifested a low incidence of increased FDG uptake and a low corresponding SUVmax, malignancy was characterized by a high incidence of increased FDG avidity and a high corresponding SUVmax, and NIID showed a high incidence of increased FDG uptake (except in the central lymph nodes) but a low corresponding SUVmax. Nevertheless, these parameters performed finitely when used separately in differentiating the cause of FUO into infection, malignancy, and NIID, and their diagnostic efficiency need to be further improved.

Preliminary studies have demonstrated that clinical parameters, such as rash, weight loss, SF, and LDH, can be combined with imaging findings to obtain a more confident diagnosis [15, 35]. Some researchers have built diagnostic models for patients with FUO, but only for particular causes or patients [9, 10, 15]. Efstathiou et al. focused on the distinction between infectious and noninfectious, and XU et al. emphasized the discrimination of bacterial infections. Wang’s research was also based on PET/CT and clinical features, but the diagnostic formula was mainly aimed at NAU patients, which was not simple and feasible in practice. Unlike previous studies that focused only on blood markers at admission, this study suggested that dynamic changes in these parameters were more conducive to a differential diagnosis, further confirming the importance of dynamic observation in the diagnosis of FUO. The indexes used to construct the models were consistent with the clinical characteristics of the corresponding diseases. There are some common clinical indicators used to identify certain etiology, such as IGRA for infection, low platelet count and splenomegaly for cancer, rash, ANA and ANCA for NIID, but they are not sufficiently sensitive and specific when used alone. Theoretically, the combined application can further improve the diagnostic performance, but there is no consensus on how to combine them. Through a large sample population in the real world, the favorable factors conducive to the etiological classification of FUO were screened out, and the weight of each index was further analyzed to obtain the formula. The AUCs of the infection, malignancy, and NIID diagnostic models were 0.89, 0.94, and 0.95, respectively, showing good discrimination ability. Similar results were obtained for the validation cohort, which verified the stability of the models. Especially for infectious diseases and NIID, the diagnostic model combined with other clinical indicators significantly improves the diagnostic efficacy of PET/CT. When PET/CT cannot provide a clear etiological diagnosis, the diagnostic models can be used to calculate the probability of each classification, rapidly and accurately distinguishing the etiologies of FUO: infectious or noninfectious, malignant or nonmalignant, and typical NIID or non-NIID.

The strengths of our study include its prospective setting, the large sample size divided into prediction and validation cohorts, the combination of clinical parameters and PET/CT imaging, and the creation of diagnostic models in a real-life clinical setting. The models were established according to PET/CT characteristics, which further improved the diagnostic efficiency of this imaging modality in FUO. The models performed satisfactorily in differentiating FUO cases, especially for malignancy and NIID. When the etiology of FUO is unclear or the clinical features are not obvious, the models can help clinicians achieve a more accurate FUO diagnosis, avoid unnecessary invasive procedures and unreasonable use of antibiotics, and reduce the rate of missed diagnosis and delayed diagnosis.

There are some limitations to this study. The single-center design likely limits the robustness of our findings. Multicenter and larger sample studies are needed to further confirm these results. Due to the wide variety of infectious diseases and obvious differences among subgroups, the sensitivity and specificity of the infection model are not adequately high. In later stages, we will further subdivide the types of infectious diseases and build more sophisticated diagnostic models. Since SUVmax is closely related to acquisition time, PET scanner, and patient metabolism and may represent only a small pixel, the use of SUVmax in this study may have limitations. More metabolic indicators, such as SUVmean or SUVpeack, can be considered in the later stage.

## Conclusions

^18^F-FDG PET/CT has a certain level of sensitivity and accuracy in the diagnosis of FUO, which can be further improved by combining clinical parameters. Diagnostic models based on PET/CT show excellent performance in the diagnosis of FUO and can be used as a reliable tool to discriminate the cause of the condition. According to the current study results on FUO patients, it is important to further investigate the application of PET/CT in diagnosing nonneoplastic diseases.

## Supplementary Information

**Additional file1**.** Fig. S1**. The ROC curve of PET/CT characteristics in diagnosing infection, malignancy, and NIID.** Table S1**. Clinical diagnosis and etiological classification in 524 patients with FUO.** Table S2**. Lesions with the most intense FDG uptake for 477 patients with FUO.

## Data Availability

The datasets generated and/or analyzed during the current study are not publicly available due to data security restrictions but are available from the corresponding author on reasonable request.

## Abbreviations

FUO: Fever of unknown origin
^18^F-FDG PET/CT: ^18^F-fluorodeoxyglucose positron emission tomography/computed tomography
NIID: Noninfectious inflammatory disease
AUC: Area under the receiver operating characteristic curve
PDCs: Potential diagnostic clues
ESR: Erythrocyte sedimentation rate
LDH: Lactate dehydrogenase
SF: Serum ferritin
IGRA: Interferon gamma release assay
SUVmax: The maximum standardized uptake value
IQR: Interquartile range
OR: Odds ratio
CI: Confidence interval
ANA: Antinuclear antibody
ANCA: Anti-neutrophil cytoplasm antibody
NAU: Nonspecific abnormal uptake
LN: Lymph node
