# Supplementary material for: Diagnostic models for fever of unknown origin based on 18F-FDG PET/CT: a prospective study in China
Source: EJNMMI Res. 2022 Oct 28;12:69. doi: 10.1186/s13550-022-00937-4 (PMC9616977; doi:10.1186/s13550-022-00937-4)
Supplement: Supplementary file 1 — Additional file1. Fig. S1. The ROC curve of PET/CT characteristics in diagnosing infection, malignancy, and NIID. Table S1. Clinical diagnosis and etiological classification in 524 patients with FUO. Table S2. Lesions with the most intense FDG uptake for 477 patients with FUO. [file 13550_2022_937_MOESM1_ESM.docx]

**Table S1.** Clinical diagnosis and etiological classification in 524 patients with FUO.

| **Etiology classification** | **Number of cases** | **Clinical diagnosis**  **（number）** |
| --- | --- | --- |
| **Infection** | 223 | *Tubercle bacillus(*52), *Epstein–Barr virus*(17), *Staphylococcus*(9), *﻿bacterium burgeri*(7), *Mycoplasma pneumoniae*(5), *Candida albicans*(5), *Cytomegalovirus*(3), *Klebsiella pneumoniae*(2), *Streptococcus*(2), *Escherichia coli*(2), *Moraxella*(2), *Histoplasma capsulatum*(2), *Leishmania*(2), *Stenotrophomonas maltophilia*(1), *Legionella*(1), *Burkholderia*(1), *Neisseria cinerea*(1), *Proteus mirabilis*(1), *Enterococcus faecium*(1), *Micrococcus luteus(*1), *Lactococcus*(1), *Hantavirus*(1), *Aspergillus flavus(*1), *Pityrisporum*(1), uncertain(102) |
| **Malignancy** | 121 | Lymphoma (77), leukemia (21), cervical cancer (4), gastric cancer (3), hepatic cellular cancer (3), breast cancer (2), lung cancer (2), mediastinal tumor (2), lymph node metastasis carcinoma(epithelial-derived) (1), multiple myeloma (1), myelodysplastic syndrome (1), duodenal carcinoma (1), colon cancer (1), prostate cancer (1), uterine cancer (1) |
| **NIID** | 109 | AOSD (56), undifferentiated connective tissue disease (12), vasculitis (12), polymyositis/dermatomyositis (4), panniculitis (3), Sjogren's syndrome (3), systemic lupus erythematosus (2), polymyalgia rheumatic (2), rheumatoid arthritis (2), EVANS syndrome (2), IgG4-related disease (2), reactive arthritis (2), autoimmune hepatitis (2), rheumatic fever (1), ulcerative colitis (1), systemic sclerosis (1), primary hemophagocytic lymphohistiocytosis (1), mixed connective tissue disease (1) |
| **Miscellaneous** | 22 | Histiocytic necrotizing lymphadenitis (15), subacute thyroiditis (5), drug allergy (2) |
| **Unknown** | 49 | Uncertain (49) |

FUO, fever of unknown origin; NIID, noninfectious inflammatory disease; AOSD, adult-onset still’s disease.

**Table S2.** Lesions with the most intense FDG uptake for 477 patients with FUO.

| **Etiology classification** | **Number of positive cases** | **The lesion with the most intense FDG uptake**  **(number)** |
| --- | --- | --- |
| **Infection** | 193 | Lymph node (77), bone marrow (24), bone or joint (21), spleen (10), lung (9), tonsil (7), liver (7), thyroid (5), nasopharynx (4), stomach (3), pons (2), pleura (2), peritoneum (2), kidney (2), colon (2), adnexa uteri (2), prostate (2), frontal lobe (1), parotid gland (1), vocal cord (1), esophagus (1), adrenal gland (1), intraperitoneal mass (1), ileum (1), rectum (1), anal canal (1), spinal cord (1), muscle (1), subcutaneous tissue (1) |
| **Malignancy** | 121 | Lymph node (47), bone marrow (21), spleen (16), nasopharynx (9), liver (8), kidney (2), adrenal gland (4), adnexa uteri (3), stomach (2), tonsil (1), thyroid (1), lung (1), colon (1), chest wall nodule (1), intraperitoneal mass (1), retroperitoneal mass (1), bone or joint (1), muscle (1) |
| **NIID** | 99 | Lymph node (38), bone marrow (21), spleen (13), bone or joint (7), tonsil (6), muscle (4), lung (3), vascular wall (1), vocal cord (1), liver (1), ileum (1), colon (1), intraperitoneal mass (1), adnexa uteri (1) |
| **Miscellaneous** | 22 | Lymph node (13), bone marrow (3), thyroid (5), adnexa uteri (1) |
| **Unknown** | 42 | Lymph node (25), bone marrow (6), spleen (3), tonsil (1), nasopharynx (1), lacrimal gland (1), saddle area (1), lung (1), adrenal gland (1), vascular wall (1), muscle (1) |

FDG, fluorodeoxyglucose; FUO, fever of unknown origin; NIID, noninfectious inflammatory disease.

Figure S1


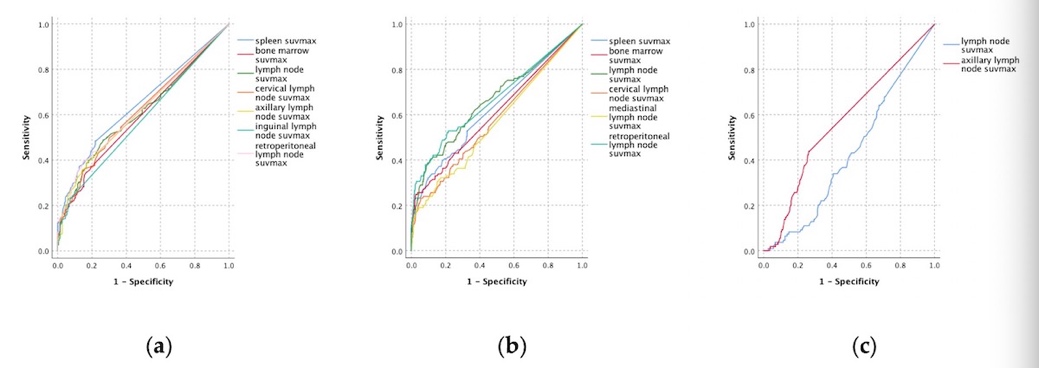


**Figure S1.** (a)The ROC curve of PET/CT in diagnosing infection. (b)The ROC curve of PET/CT in diagnosing malignancy. (c)The ROC curve of PET/CT in diagnosing NIID.
